# Composites from Recycled Polyolefin and Waste Plant Biomass with Potential Uses in Electrical Insulation Applications

**DOI:** 10.3390/ma19071415

**Published:** 2026-04-01

**Authors:** Mihaela Aradoaei, Romeo Cristian Ciobanu, Sebastian Teodor Aradoaei, Rolland Luigi Eva, Alina Ruxandra Caramitu, Adriana Mariana Bors

**Affiliations:** 1 Department of Electrical Measurements and Materials, Gheorghe Asachi Technical University, 700050 Iasi, Romania; mihaela.aradoaei@academic.tuiasi.ro (M.A.); arsete@tuiasi.ro (S.T.A.); darkrollandluigi@yahoo.com (R.L.E.); 2 National Institute for Research and Development in Electrical Engineering—ICPE-CA, 030138 Bucharest, Romania; 3 National Research and Development Institute for Optoelectronics INOE 2000, Subsidiary Research Institute for Hydraulics and Pneumatics IHP, 040558 Bucharest, Romania; bors.ihp@fluidas.ro

**Keywords:** recycled polyethylene, recycled polypropylene, poplar seeds, vegetable peels, polymer composites, dielectric properties, electrical insulation applications

## Abstract

This research investigates novel polymeric composite materials made from recycled polyolefin and waste plant biomass (poplar seeds and vegetable peels), which have potential applications in the relatively unexplored field of electrical insulation. For composites made from poplar seeds with low density polyethylene matrix, the structure appears more uniform, even with increased biomass content, in contrast to those utilizing high density polyethylene matrix, which displays notable heterogeneous areas where the polymer appears separated from the fibrous network at higher biomass levels. Concerning the composites of vegetable peels with high density polyethylene matrix, the fragments of vegetable peels are clearly recognizable, and their bond to the polymer matrix appears weaker. When incorporating vegetable peels into the polypropylene matrix, it results in a better distribution of the vegetable peel fragments within the polymer matrix, as well as enhanced structural homogeneity. Overall, the incorporation of biomass reduces the Shore hardness measurement for every polymer matrix. Regarding tear resistance, the inclusion of biomass reduces the values only for low density polyethylene with poplar seeds. For both high density polyethylene and polypropylene, regardless of the biomass type, the property seems to enhance marginally with the addition of biomass. The primary advantage of utilizing these composites is that their water absorption rate is at least twice as low as that of transformer board, while still offering a similar capacity for absorbing transformer oil. All composite types exceeded the minimum required threshold of 70 °C for service exposure, and adhered to insulation class A, similar to cellulose-based insulations. The addition of cellulose to polyolefin composites appears to slightly improve their breakdown strength. The conductivity for this type of composite is at least three times lower than that of cellulose insulation materials, rendering them beneficial for applications in electrical engineering as potential substitutes for cellulose-based materials in multiple electrical insulation uses, e.g., for insulating low voltage electrical machines, as well as serving as a substitute for pressboard in transformers. Additionally, their thermoplastic properties offer enhanced processing versatility, opening up new opportunities for electrical engineering technology, especially with regard to electrical insulation recyclability in the context of a circular economy.

## 1. Introduction

Plant biomass waste comprises organic byproducts from agriculture and/or forestry, including forest or crop residues. Such resources may decrease dependence on fossil fuels, handle waste in an eco-friendly manner, and although they may release CO_2_ during transformation, are viewed as carbon-neutral since the carbon was previously absorbed by the plants. Their use also lessens the strain on landfills. In the last decade, there has been significant attention on eco-friendly technology and sustainable practices through the use of plant biomass waste. Even if their classical use related mainly to renewable energy (electricity, heat, fuel) via combustion, gasification, or anaerobic digestion [1,2,3,4], the new trend is to transform them into innovative products in the manufacturing and assembly sector. The concept of manufacturing thermoplastic composites from recycled polyolefin and waste plant biomass is emerging as a revolutionary substitute for conventional plastics. In contrast to traditional plastics derived from fossil fuels, these composites leverage the prominent impact of cellulose fibers, with modifications in parameters and properties, making them suitable for diverse applications. This makes products lightweight and easy to handle, adding the mechanical strength, rigidity, and thermal stability of the composite, with the additional resistance to moisture and UV rays, making them suitable even for outdoor applications.

The production of such composites uses less energy compared to traditional plastics, and aside from that, their manufacture does not release greenhouse gases, which makes them the most eco-friendly plastics. Readily accessible plant biomass waste renders the bio-composite manufacture highly economical. Given the extensive range of organic byproducts from agriculture and/or forestry, the current literature is correspondingly varied in terms of technologies and utilized bio-resources [5,6]. Such examples of experimental thermoplastic composites are related to the use of mixed agricultural fibers [7], millet husk crop-residue [8], coffee husk [9,10,11], banana/betel nut husk [12], kapok husk [13], olive husk [14,15], corn husk [16,17], and rice husk [18,19], with some of them using polyethylene [10], polypropylene [13], polyvinylchloride [14], or polystyrene [19] as polymer matrix. Utilizing fiber sourced from plant biomass waste in the production of these composites is seen as highly beneficial, mainly by potentially replacing, for instance, wood flour used in wood plastic composite (WPC) formulations. Typically, WPCs are made with wood flour usage weights reaching as high as 50%; a partial replacement of wood flour with fiber sourced from plant biomass waste is presented in [20,21,22]. The total replacement of wood flour with cellulose and lignin derivatives in thermoplastic composites proved advantageous, partly due to enhanced mechanical properties and eliminating the issue of residual moisture in wood flour/saw dust during WPC production [23,24].

As agriculture is based on extensively cultivated crops, a steady availability of plant biomass waste is expected to encourage bio-composite manufacturing. Aside from that, the increasing need for sustainable products creates a strong market for such bio-composites. This relies on the principle that WPC products still show an expanding market with numerous potential uses, with an increase from USD 8.91 billion in 2025 to USD 9.67 billion in 2026, and further projected to attain USD 14.54 billion by 2031 and an 8.49% CAGR from 2026 to 2031 [25]. As potential substitutes for WPCs, the bio-composites may be consistently incorporated for use in multiple industries as specific materials for construction and manufacturing, e.g., in the automotive industry for producing interior panels, dashboards, and various other components, in the building sector for insulation, siding, etc., and also for producing containers, or for a variety of consumer goods such as furniture, kitchen utensils, and home goods. Bio-composites offer flexibility, being customizable and featuring an attractive design.

Nonetheless, despite growth opportunities, the bio-composites sector also encounters several obstacles. The main technological risk includes scalability issues and acceptance in the market. However, ongoing research in the field does provide advantageous opportunities for effectively overcoming such obstacles.

Historically, natural fibers and fibrous materials have been thoroughly researched for use in dielectric applications. Natural fibers are favored as dielectric materials due to their hydrophilic nature, reasonable mechanical and dielectric characteristics, and the ability to be formed into products of different shapes. However, despite their variety, a unified examination of the dielectric characteristics of thermoplastic bio-composites has not been performed to finally enable a dependable implementation in the field of electrical engineering. Timely studies in the area of electrical characteristics, as presented in [23,24,26,27], have revealed just a limited perspective on the intricate problem of the potential applications of thermoplastic bio-composites as insulating materials, without associating plant biomass waste features with a specific use in electrical engineering, such as possibly replacing current insulations with eco-friendly alternatives. Conversely, to the best of our knowledge, the current literature does not contain studies linking plant biomass waste with recycled plastic matrices for the production of bio-composites within the larger context of a circular economy. The purpose, and, at the same time, the novelty of this research, was to obtain new composite materials of recycled polyolefin and waste plant biomass (here, poplar seeds and vegetable peels—zucchini, cucumber and aubergine) with potential uses in electrical insulation applications, a less-investigated domain at present.

## 2. Materials and Methods

### 2.1. Materials Used

The raw materials utilized to produce the polymer composite materials included:

(1) Matrix: recycled low and high density polyethylene (LDPEr and HDPEr) and polypropylene (PPr) sourced from electronic waste (All Green SRL, Iasi, Romania), using the technological method of processing detailed in [28,29]. The XRF analysis and properties of recycled polyolefins from electronic waste are presented in [30].
(2) Poplar seeds with fibers (PS) present a fibrous framework around the small seed, are hydrophobic and oleophilic, and contain 62.07% cellulose, 17.04% lignin, and 2.5% ash. They were collected in June (Technical University Iasi, Romania), utilizing leaf blowers, and had a high level of purity, but were manually inspected and ultimately cleaned from debris; their properties resemble those outlined in [31]. They showed a maximum humidity of 25%.
(3) Mixed vegetable peels—zucchini, cucumber and aubergine (VP)—were collected in a dedicated container associated with a large canteen kitchen to prevent mixing with other organic waste (Technical University Iasi, Romania). They presented a maximum humidity of 75%.
(4) Maleic anhydride grafted polyethylene/polypropylene (PEAM/PPAM) (Nanjing Feiteng Chemical Co. Ltd., Nanjing, China) were used as coupling agents for particle surface functionalization [32], 3.5% (wt%) in all samples.
(5) Licocene™ PE MA 4351 fine grain (Clariant AG, Muttenz, Switzerland) was added as compatibilizer and dispersing agent, 1.5% (wt%) in all samples.

### 2.2. Technological Methodology

The authors have used a novel and eco-friendly sterilization method for the waste plant biomass utilizing microwave radiation in enclosed air-circulation ovens. The samples underwent a joint drying–sterilization procedure, conducted until the material exhibited minimal remaining moisture. Microwave radiation exposure was conducted at a power level of 800 × 10^3^ W/kg for a duration of 0.5 min in the case of poplar seeds, and just over 1 min for vegetable peels, to assure a residual humidity under 8%, as described in [23]. Further, the raw materials were submitted to two-stage dry grinding/milling, resulting in a mixed powder with final dimensions below 1 mm, utilizing a high-efficiency plastic shredder machine (Henan Gomine Industrial Technology Co., Zhengzhou, China) and then a pulverizer-milling machine (Jiangsu Xinhe Intelligent Equipment Co., Ltd., Taizhou, China) to form a micron-sized powder.

The polymer composite materials were produced in two phases; namely, composite pellets were formed through extrusion using a laboratory twin-screw extruder Lab-Compounder KETSE 20/40 (Brabender, Duisburg, Germany), and then subjected to a melt injection process with a Dr. Boy 35A injection-molding machine (Dr. Boy GmbH & Co. KG, Neustadt-Fernthal, Germany) to create testing samples. Concerning the injection process, working temperatures between 170 and 230 °C and clamping forces of 138–155 kN were utilized to produce samples of 2 ± 0.1 mm in thickness, with shapes related to the needs of the mechanical and dielectric testing methods.

### 2.3. Samples Description

The samples description is presented in Table 1.

Due to the anticipated potential application in electrical engineering, the highest addition of biomass was considered as 30% for both scenarios, even though the intended uses may differ, and theoretically, the experiments would extend the biomass content up to 50%. In line with the anticipated application, thin and flexible insulation for electrical machines, PS is a resource more difficult to access, composites that are more flexible and have lower density are expected, and only LDPEr is suitable as a matrix. In the situation of inexpensive and readily available PV, thick and more rigid composites are anticipated, with an application akin to pressboard for transformers, where only HDPE and PP matrices are suitable. That is why PPr was not further discussed with PS, and conversely, LDPEr was not discussed with VP, even if initially the research generated every possible combination, meaning any matrix containing both PS and VP. This is why Table 1 was also condensed—to avoid overwhelming the experimental descriptions.

### 2.4. Characterization Methods

(i) Field emission and focused ion beam scanning electron microscopy (SEM) were conducted using a Quanta FEG 250 (Thermo Fisher Scientific Inc., Waltham, MA, USA). The method of analysis employed water vapor to safeguard the samples from harm, and the occurrence of SEM charging was greatly diminished because of the low vacuum in the specimen chamber.
(ii) The hydrostatic density was evaluated using an XS204 Analytical Balance (Mettler-Toledo, Columbus, OH, USA). The tests were performed at a temperature of 21 °C, and the density was calculated as the average of three successive repeated measurements.
(iii) Shore hardness measurements were obtained using a commercial Shore “D” digital durometer, calculated as the average of five readings, in accordance with [33].
(iv) The apparatus used to assess the mechanical properties was a specialized computer-operated PULL-2000KG Universal Tensile Testing Machine (Lisun Group, Qiantong, Zhejiang, China), which has a minimum nominal force of 20 kN, enabling the evaluation of tensile strength and elongation in accordance with [33].
(v) The level of swelling was assessed by measuring the change in mass of the samples at specified immersion times, using the XS204 Analytical Balance (Mettler Toledo, Columbus, OH, USA), as described in [24,34].
(vi) The dielectric characteristics were assessed using a Broadband Dielectric Spectrometer (Novocontrol GMBH, Montabaur, Germany) that featured an Alpha frequency response analyzer and a Quattro temperature controller, and equipped with specialized measurement cells that can operate up to 40 GHz.

## 3. Results and Discussion

### 3.1. Structural Analysis—SEM

**Figure 1:**
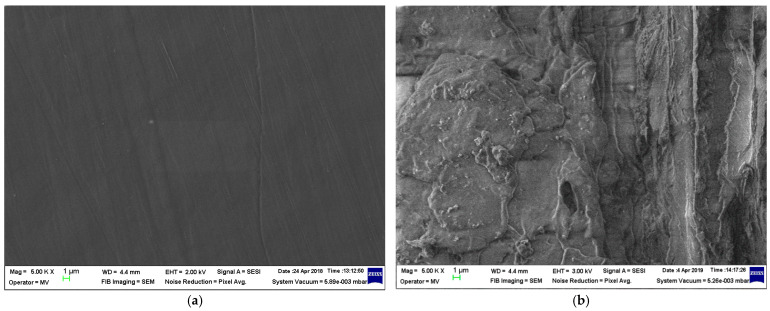

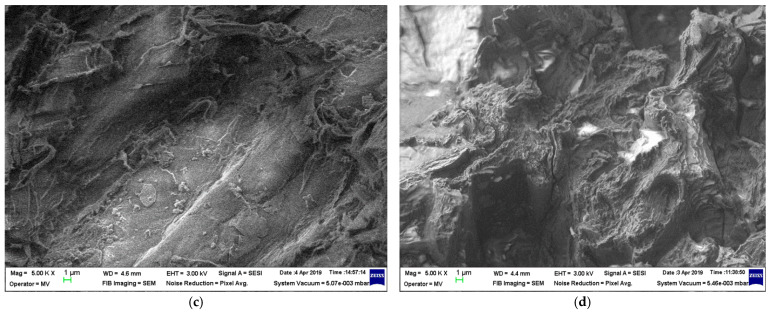
Micrographs of (**a**) LDPEr; (**b**) L-PS1; (**c**) L-PS2; and (**d**) L-PS3.

**Figure 2:**
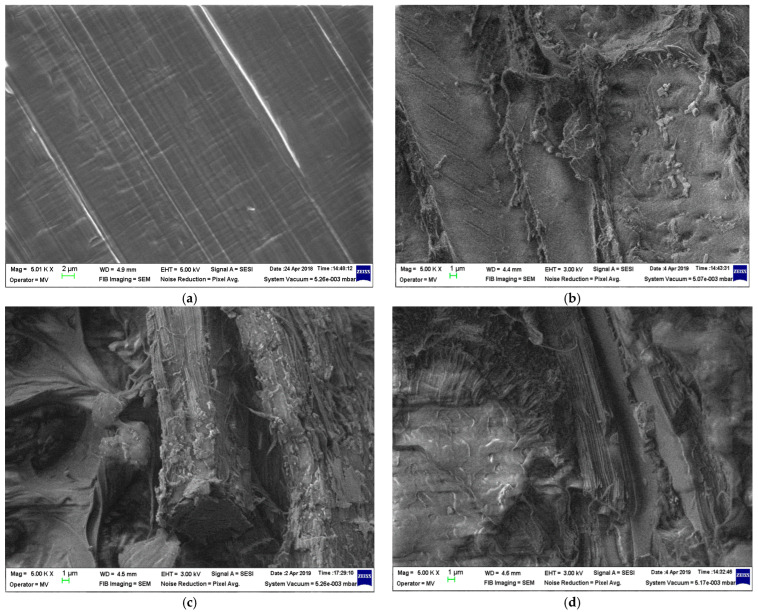
Micrographs of (**a**) HDPEr; (**b**) H-PS1; (**c**) H-PS2; and (**d**) H-PS3.

**Figure 3:**
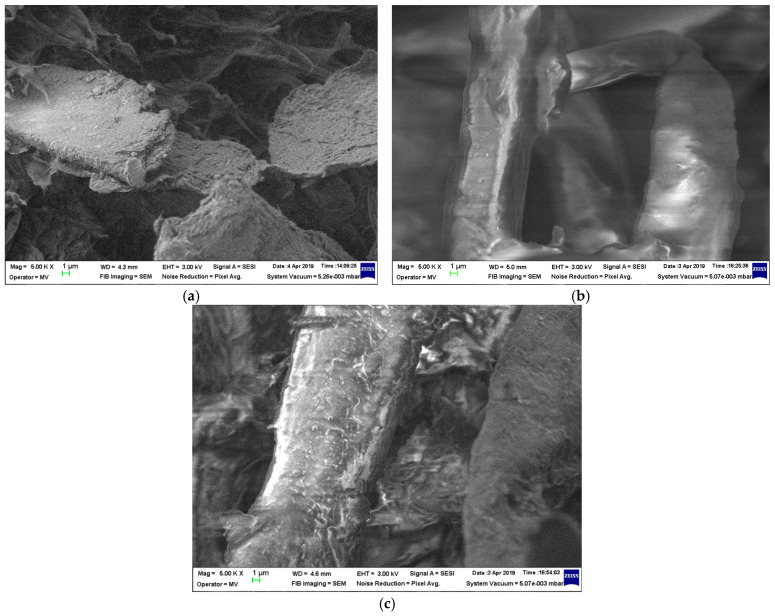
Micrographs of (**a**) H-VP1; (**b**) H-VP2; and (**c**) H-VP3.

**Figure 4:**
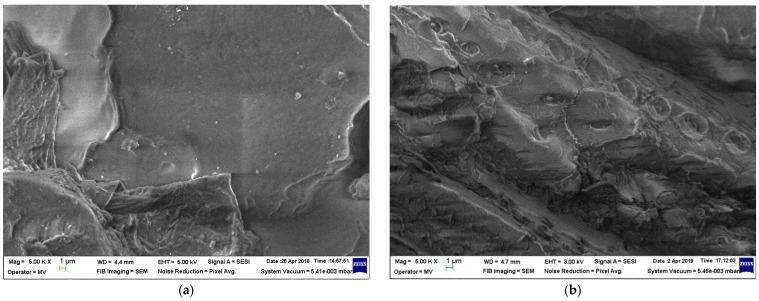

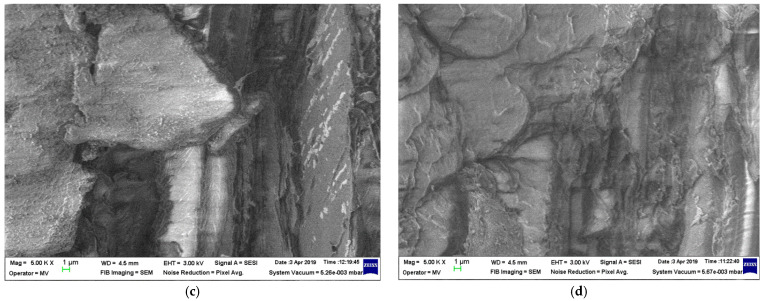
Micrographs of (**a**) PPr; (**b**) P-VP1; (**c**) P-VP2; and (**d**) P-VP3.

The structural analyses of samples are presented in Figure 1, Figure 2, Figure 3 and Figure 4 at 5000 magnification.

In Figure 1 and Figure 2, a fine structure of rests in the fibrous framework incorporated within the polymer matrix, and small seed rests, can be identified. For the LDPEr matrix, the structure appears to be more uniform even with increased biomass content, in contrast to the composite with the HDPEr matrix, which shows large heterogeneous areas where the polymer seems to be disconnected from the fibrous network at higher biomass levels.

The same heterogeneous aspect is noticed in Figure 3, which is similarly linked to the utilization of the HDPEr matrix. In this case, the fragments of vegetable peels are clearly recognizable, yet their bond to the polymer matrix is less strong, given that their inclusion looks more like a heterogeneous mixture at any biomass levels. Finally, as regards Figure 4, the assessment of the structural performance of these composite materials indicates that even for larger content of biomass, the use of PPr results in better dispersion within the polymer matrix. In summary, regarding the optimal structure and bonding of composites, the composites of poplar seeds with LDPE and of vegetable peels with PPr matrix can be recommended.

### 3.2. Mechanical Properties

In conjunction with the information provided in Section 2.4, the tear resistance, elongation at break, and Young’s modulus were determined as indicated in [34], and the CHARPY impact test (type 1 specimens, type A notch, pendulum) according to [35]. The results are presented in Table 2.

Overall, the inclusion of biomass slightly reduces the Shore hardness values for all polymer matrices. Similar behavior presents with regard to the elongation. As regards shock resistance, the inclusion of biomass slightly reduces the values, but only in the case of HDPEr and PPr. In the case of the LDPEr matrix, the shock resistance values slightly increase with the progressive addition of poplar seeds, taking into account that typically LDPE exhibits low resistance and the incorporation of reinforcements enhances its properties. As regards tear resistance, the inclusion of biomass slightly reduces the values, but only in the case of LDPEr with poplar seeds. In the case of HDPEr and PPr, regardless of the biomass type, the tear resistance slightly improves with the addition of biomass, an aspect in line with the general behavior of reinforced polyolefins. The highest value tear resistance is present in the composites with fragments of vegetable peels, regardless of the matrix used—an aspect that can be associated with the findings in Figure 3 and Figure 4, where the biomass fragments are bigger in comparison to those derived from the poplar seeds.

### 3.3. Physical-Thermal Properties

In relation to the information shared in Section 2.4, the VICAT softening temperature (5 kg), method A 50, was determined as indicated in [36], the molding shrinkage as in [37], and the melt flow index (190 °C; 5 kg) according to [38]. The results are displayed in Table 3.

It can be noted that the inclusion of biomass slightly reduces the values of density, melt flow index and VICAT softening temperature for all polymer matrices. The molding shrinkage remains about two in all circumstances, similar to the one for polyolefins, and practically being uninfluenced by the biomass addition. Similarly, the melting point is defined only by the polymer matrix used, being higher in the case of PPr, where also the fluidity index presents the highest values. It is obvious that these characteristics do not have a direct connection to the type or percentage of biomass utilized, but provide important information regarding the processability of composites to produce components for electrical engineering applications.

### 3.4. Liquid Absorption Features

In order that new composite materials of recycled polyolefin and waste plant biomass are to be successful substituents for cellulose-based insulation materials, they need to provide minimal water absorption and a reasonable transformer oil absorption capacity. Accordingly, the experiments were conducted up to an immersion duration of 576 h and utilized water and transformer oil. Liquid absorption characteristics were determined as presented in [23,39]. The experimental results are shown in Table 4 and Table 5.

The purpose of this research was to obtain new composite materials of recycled polyolefin and waste plant biomass with potential uses in electrical insulation applications, the aim being to potentially replace more costly and resource-consuming insulations based on cellulose. The insulation pressboard for transformers [40], classified as Class A (105 °C), is made from unbleached cellulose sourced from long-fibered coniferous wood, possessing chemical purity, mechanical strength, and ideal oil absorption properties. The pressboard is mainly utilized in oil-filled power and distribution transformers, but it might also be used, in a slenderer shape, for insulation in large electric machines and/or capacitors, in spite of its limited flexibility.

The results presented above are relevant when comparing composite materials to insulation materials based on cellulose, e.g., the transformer board can take in water up to over 40% relative to its initial weight, and when immersed in transformer oil, it achieves about 15% absorption rate after approximately 240 h, reaching a saturation limit.

In our case, the composite materials of recycled polyolefin and waste plant biomass tend to reach saturation after 504 h in the case of an addition of 30% biomass, and even later, after 576 h, in the case of a lower addition of biomass, due to the influence of matrix and internal architecture. It is evident that when assessing the behavior of the transformer board with the samples L-PS2, L-PS3, H-PS2, H-VP2, P-VP2 and P-VP3, the absorption rate for transformer oil nears 15% in all cases, mainly at a higher content of biomass in the case of composites based on LDPEr and PPr. Nonetheless, the primary advantage of utilizing these composites is that their water absorption rate is at least twice as low compared to transformer board, yet they offer a similar transformer oil absorption capacity.

With regards to water absorption, the presence of the polymer matrix drastically limits this parameter, as compared to classical paper-based electrical insulation. Following a detailed examination of the absorption properties of both water and transformer oil, and correlating this with the mechanical features and material structure highlighted by the SEM images (Figure 1, Figure 2, Figure 3 and Figure 4), it is logical to suggest that materials with a HDPEr matrix require no further analysis, allowing for more attention to be directed towards those based on LDPEr and PPr. These composites have a higher content of biomass, are more feasible and cost-efficient for electrical engineering applications, e.g., the instance of LDPEr-based composites for insulating low voltage electrical machines, and PPr-based composites can serve as a substitute for pressboard in transformers.

### 3.5. Dielectric Characteristics

In order that new composite materials of recycled polyolefin and waste plant biomass be successful substituents for cellulose-based insulation materials, their dielectric features must be in line with those offered by the pressboard insulation, especially dielectric strength ≥ 40 kV/mm.

Dielectric strength was determined according to [41], and the results are presented in Table 6.

It was generally noticed that the breakdown strength of polyolefin composites, with the addition of cellulose, may demonstrate a low improvement [42], and the findings shown above align with these assumptions. The resulting values enable all composites to function as insulating materials since all values exceed the minimum required dielectric strength, providing the benefit of thinner insulation, along with more favorable costs.

**Figure 5:**
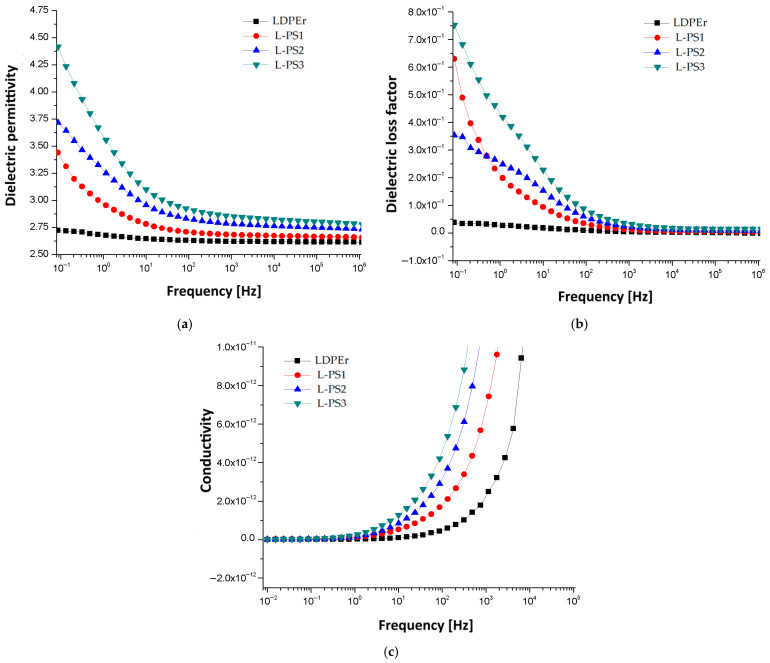
Dielectric properties of composites with LDPEr matrix: (**a**) dielectric permittivity ε′; (**b**) dielectric loss factor ε″; and (**c**) conductivity [S/cm].

**Figure 6:**
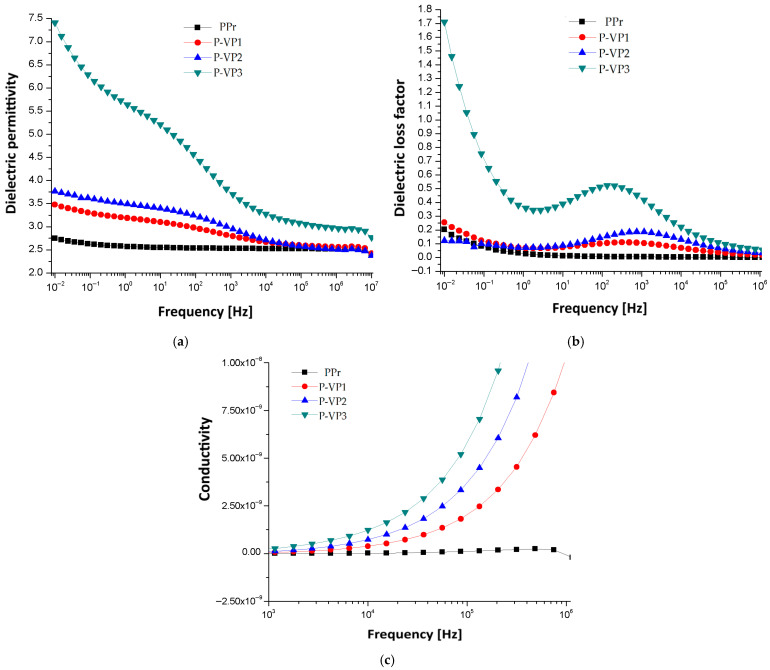
Dielectric properties of composites with PPr matrix: (**a**) dielectric permittivity ε′; (**b**) dielectric loss factor ε″; and (**c**) conductivity [S/cm].

The ultimate conclusion regarding the most suitable combination should be made by examining the dielectric properties of composites: (a) dielectric permittivity ε′; (b) dielectric loss factor ε″; and (c) conductivity [S/cm]. The main results are summarily presented in Figure 5 and Figure 6.

For both polymer matrices, the dielectric permittivity exhibits the well-known classical decrease with increasing frequency, and, for each type of biomass, the permittivity rises at lower frequencies as the percentage increases, due to the increase in interfacial polarization. It is clear that the increase is larger in the case of composites with larger biomass fragments in their structure; at larger biomass contents, the case of vegetable peels in PPr Figure 6a, compared to the case of poplar seeds in LDPEr, Figure 5a, is illustrative. Similarly, the presence of interfacial polarization at lower frequencies is noticed in the case of dielectric loss factor for all presented specimens. It is also much larger in the case of composites with larger biomass fragments in their structure, as in the case of vegetable peels in PPr, Figure 6b, with poplar seeds in LDPEr, Figure 5b. Moreover, a distinct dipolar polarization can be seen in the samples P-VP1 to P-VP3 within the 10^2^–10^4^ Hz frequency range, attributed to the polarization effect of polypropylene. It is notable that the permittivity and dielectric loss values for this kind of composite are somewhat lower than those of insulating cellulose materials, which is advantageous for their application [42].

In all circumstances, conductivity, exhibited in Figure 5c and Figure 6c, rises at frequencies over 10^4^ Hz as the biomass percentage increases. However, it should be noted that the conductivity values for this type of composite are at least three times lower than the conductivity of insulating cellulose materials, which is beneficial for utilizing these composites in electrical engineering applications [42].

### 3.6. Thermal Stability and Insulation Class Evaluation

**Figure 7:**
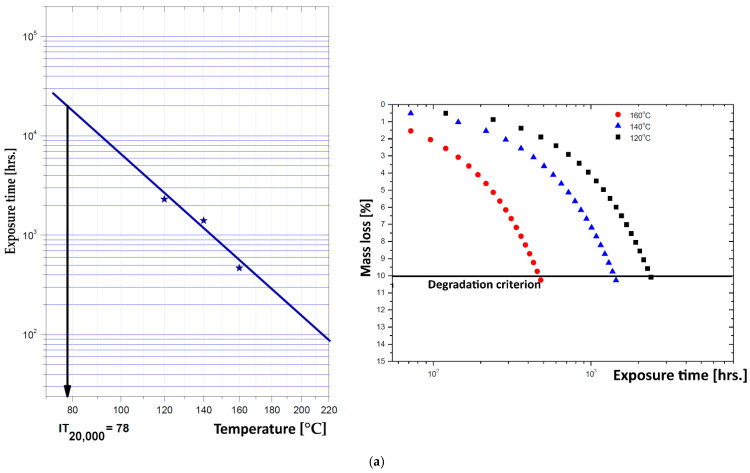

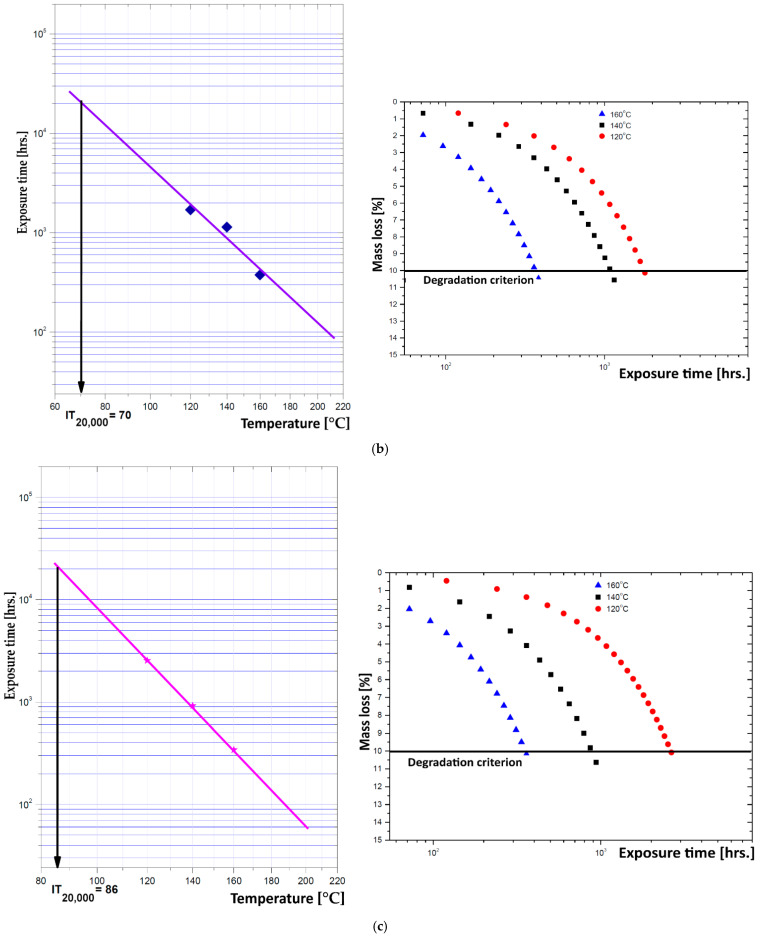

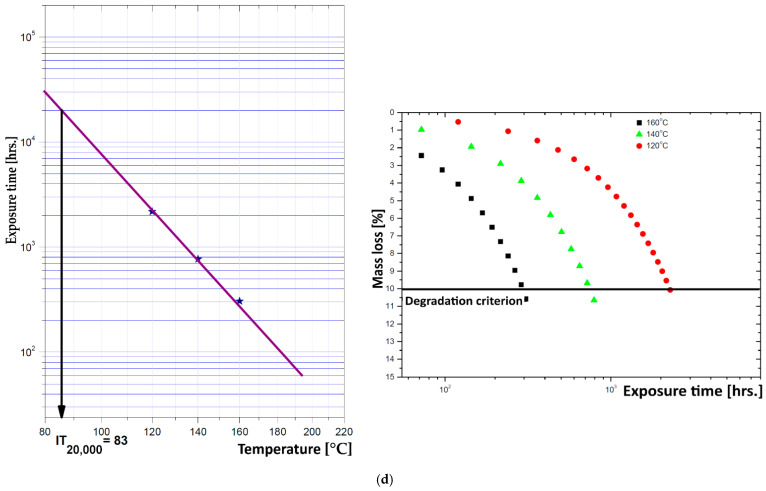
Arrhenius diagrams and Mass loss characteristics for: (**a**) L-PS1; (**b**) L-PS3; (**c**) P-VP1; (**d**) P-VP3.

In order to determine the thermal stability and insulation class, an accelerated aging test was performed on these composite materials, with a minimum exposure temperature of 120 °C and a maximum of 160 °C, with the exposure durations akin to those used in [43]. To analyze the results from these tests, the Arrhenius equation was utilized, which defines the relationship between the chemical degradation reaction coefficient and the temperature, tailored to reflect the correlation between the material’s lifespan and temperature. The selected degradation criterion for these materials was mass loss, assuming that the materials reach end of life at a 10% mass loss (corresponding to the temperature index at 20,000 h of operation). Figure 7 shows the mass loss characteristics with the degradation criterion for each temperature of exposure, and the Arrhenius diagrams with the index at 20,000 h of operation, for each sample.

As expected, due to a more advanced degradation of biomass content vs. temperature, the IT_20000_ factor is lower for higher biomass content for both polymer matrices used, Figure 7b,d. Conversely, the composites featuring a PPr matrix, Figure 7c,d, demonstrate greater thermal stability, exhibiting an IT_20000_ value that is at least 10 °C higher in comparison to LDPE-based composites. Nonetheless, in every instance, the minimum accepted threshold of 70 °C for service exposure in the intended applications was exceeded. The acquired values relate to the thermal exposure of materials per the electrical engineering standard, particularly concerning the insulation class [43,44]; in this case, they were aligned with insulation class A (usage limit of 105 °C, exposure limit of 70 °C for lifespan evaluation—in this scenario exceeding 20,000 h for all samples), similar to cellulose-based insulations/transformer board.

Taking into account the characteristics of polyolefin and biomass composites presented above, we may conclude that they present similar mechanical properties, a higher dielectric strength, a much lower electric conductivity, a similar insulation class A (105 °C), a good compatibility and reasonable absorption characteristics of transformer oil, so they can be successful substitution candidates for cellulose-based materials in several applications in electrical insulation. In addition, due to their thermoplastic features, they offer more flexibility in processing, providing new opportunities for electrical engineering technology.

Gathering and correlating all technical data, we can recommend P-VP2 and P-VP3 composites as substitutes for transformer board, with thicknesses in the range of 0.5–30 mm (special sizes and shapes can be customized), and L-PS2 and L-PS3 for insulations in large low-voltage electrical machines, with thicknesses under 0.5mm, being more flexible and more reliable compared to cellulose-based insulation.

### 3.7. Aspects Concerning Flammability and Fire Resistance

Thermal class (insulation class), as stated earlier, indicates the highest operating temperature that the material can endure for a specified duration. Fire retardancy additionally outlines a material’s capacity to withstand ignition, extinguish itself, and prevent flame propagation when exposed to high temperatures, overloads, or arcing. Electrical insulation requires specific fire resistance ratings to be deemed effective in numerous applications, especially in construction and industrial environments where safety regulations are enforced, though these applications primarily pertain to cables. However, electrical insulation for transformers and machines needs to possess particular fire resistance or flame retardancy ratings to be considered safe, dependable, and effective. The standards outlined in these directions are very recent, reflecting the current focus on the safe use of such equipment [44,45,46]. In this context, evaluating the fire resistance or flame retardancy of the materials mentioned in the study is important, in line with the tests in [44] on transformers (even if focusing mainly on dry transformers) and in [45] on electrical machines. In transformers filled with oil, mineral oil is combustible, but the solid insulation (made from cellulose) inside should not increase the risk. The common standard for flammability testing is UL 94 [47], and for limiting oxygen index, it can be found in [48]. Furthermore, to assess the heat propagation capability, the thermal conductivity of composites was examined using a LFA 447 Nanoflash apparatus (Netzsch, Selb, Germany), with the testing method as specified in [49]. Limited research exists on the flammability of cellulose-polymer composites; however, in [50], it was discovered that cellulose fibers helped to create an integrated fibrous-intumescent char structure with improved barrier properties.

The composites with biomass achieved a slow-burning horizontal rating (UL-94 HB), but did not pass the vertical test (UL-94 V-0/V-1/V-2) as the burning material dropped and ignited fragments, with low smoke development. We should note that these are still flammable materials (LOI < 25%), although their performance is distinctly superior to that of polymer matrices, which received a “No Rating” (NC) because they ignite rapidly when subjected to flames. The results are presented in Table 7. The thermal conductivity values align with findings for comparable materials reported in [51], but show an increase at higher values with the addition of biomass, as observed in [52].

Upon examining these parameters, we found that incorporating biomass in polyolefins slightly enhances both flammability and fire resistance, a noteworthy technical finding. Essentially, with a low amount of biomass, the composites still ignited easily in regular atmospheric air, which has about 21% oxygen, but at the highest biomass levels, the performance is noticeably improved, e.g., in the case of H-VP3 and P-VP3, the oxygen threshold of 21% was surpassed at 22%. The phenomenon is attributed to the ability of biomass to enhance the structural integrity of composites while not substantially raising thermal conductivity due to a greater lignin concentration compared to paper for electrical purposes, as lignin reduces cellulose flammability. The advantage of lignin for enhancing the fire safety of polyolefins is an innovative concept, as briefly noted in [53]. Conversely, the effect could also be explained by the weak interfacial adhesion and lower inherent thermal conductivity of biomass when organized randomly. It is recognized that polyolefins utilized in cables frequently require flame-retardant modifications, such as the application of phosphorus or halogen-containing substances. However, if necessary, the mentioned polyolefin–biomass composites can be enhanced with regard to flammability through the inclusion of comparable compounds.

In the context of a circular economy aiming to enhance the recyclability of electrical insulation, polyolefin–biomass composites could provide a dependable solution, considering that cellulose-based electrical insulation, when immersed in transformer oil and similar substances, is virtually non-recyclable. These composites, owing to their thermoplastic properties, can be recycled using similar thermoplastic methods.

## 4. Conclusions

In this work, new composite materials of recycled polyolefin and waste plant biomass (poplar seeds and vegetable peels—zucchini, cucumber and aubergine, respectively) were obtained and tested, with potential identified uses in electrical insulation applications.

In the case of composites of poplar seeds with a LDPEr matrix, the structure seems more homogenous, even with higher biomass content, unlike the ones based on a HDPEr matrix, which reveals significant heterogeneous regions where the polymer appears detached from the fibrous network at elevated biomass levels. With regard to the composites of vegetable peels with a HDPEr matrix, the fragments of vegetable peels are distinctly identifiable, and their adhesion to the polymer matrix seems weaker, as their presence resembles a heterogeneous mixture across all biomass levels. Ultimately, the incorporation of vegetable peels within a PPr matrix leads to an improved distribution of the fragments of vegetable peels within the polymer matrix, and to an improved homogeneity of the structure.

In general, the addition of biomass lowers the Shore hardness values for each polymer matrix. Analogous behavior occurs with the elongation at break. Concerning the CHARPY impact test, adding biomass slightly lowers the values solely for HDPEr and PPr. For the LDPE matrix, the CHARPY impact test values observe a slight rise with the gradual addition of poplar seeds. Concerning tear resistance, the addition of biomass decreases the values solely for LDPEr with poplar seeds. For both HDPEr and PPr, irrespective of the biomass type, the property appears to improve slightly with biomass addition, consistent with the typical behavior of polyolefins reinforced with additives.

The main benefit of using these composites is that their water absorption rate is at least two times lower than that of transformer board, while providing a comparable capacity for absorbing transformer oil.

It was observed that the breakdown strength of polyolefin composites, when cellulose is added, show a slight enhancement; the resulting values allow all composites to serve as insulating materials as each value surpasses the minimum dielectric strength needed, offering the advantage of reduced insulation thickness and improved cost-effectiveness.

It is important to highlight that the conductivity for this category of composite is at least three times less than that of homolog cellulose insulation materials, making them advantageous for use in electrical engineering applications.

The minimum required threshold of 70 °C for service exposure in the intended applications was surpassed by all composite types. They were consistent with insulation class A, akin to cellulose-based insulations.

Considering the properties of polyolefin–biomass composites mentioned in the paper, we can conclude that they exhibit comparable mechanical properties, enhanced dielectric strength, significantly reduced electrical conductivity, a similar insulation class, and adequate transformer oil absorption characteristics, making them viable alternatives to cellulose-based materials for various electrical insulation applications. The composites based on LDPEr and PPr, with high contents of biomass, are feasible and cost-efficient for two potential electrical engineering applications, e.g., the instance of LDPEr-based composites for insulating low-voltage electrical machines, as well as the instance of PPr-based composites serving as a substitute for pressboard in transformers.

Furthermore, their thermoplastic characteristics provide greater processing flexibility, creating new possibilities for electrical engineering technology. Within the framework of a circular economy and focusing on improving the recyclability of electrical insulation, polyolefin–biomass composites may offer a reliable solution, especially since cellulose-based electrical insulation is nearly non-recyclable.

## Figures and Tables

**Table 1:** Samples description (addition in wt%).

Samples	Matrix/Biomass	Biomass Addition
LDPEr	LDPEr	0
L-PS1	LDPEr/PS	10
L-PS2	LDPEr/PS	20
L-PS3	LDPEr/PS	30
HDPEr	HDPEr	0
H-PS1	HDPEr/PS	10
H-PS2	HDPEr/PS	20
H-PS3	HDPEr/PS	30
H-VP1	HDPEr/VP	10
H-VP2	HDPEr/VP	20
H-VP3	HDPEr/VP	30
PPr	PPr	0
P-VP1	PPr/VP	10
P-VP2	PPr/VP	20
P-VP3	PPr/VP	30

**Table 2:** Mechanical properties (error < 5%).

Samples	Shore Hardness Shore D [ShD]	CHARPY Impact Test [KJ/m^2^]	Tear Resistance [MPa]	Elongation [%]	Young’s Modulus [MPa]
LDPEr	31	42	19.3	16	255
L-PS1	26	43	18.3	11	268
L-PS2	25	44	18.0	6	276
L-PS3	24	45	17.6	4	285
HDPEr	42	58	25	4.1	131
H-PS1	31	49	26	3.6	120
H-PS2	30	46	27	3.4	116
H-PS3	29	41	29	3.2	150
H-VP1	30	53	28	3.1	140
H-VP2	29	49	35	2.9	137
H-VP3	28	43	33	2.7	131
PPr	46	52	30	3.2	156
P-VP1	32	45	31	2.6	153
P-VP2	31	43	33	2.4	146
P-VP3	29	41	37	2.3	131

**Table 3:** Physical-thermal properties (error < 5%).

Samples	Density [g/cm^3^]	Melt Flow Index [g/10 min]	Melting Point [°C]	VICAT Softening Temperature [°C]	Molding Shrinkage [%]
LDPEr	0.923	4.62	115–120	49	2.15
L-PS1	0.880	4.31	125–135	47	2.1
L-PS2	0.835	4.22	125–135	46	2
L-PS3	0.785	4.15	125–135	45	2
HDPEr	0.958	7.59	125–130	69	2.1
H-PS1	0.870	6.88	135–145	66	2.07
H-PS2	0.823	5.64	135–145	64	2
H-PS3	0.778	4.91	135–145	62	2
H-VP1	0.865	6.34	135–145	64	2.05
H-VP2	0.811	5.24	135–145	62	2
H-VP3	0.762	4.17	135–145	59	2
PPr	0.897	16.04	160–165	89	2.05
P-VP1	0.858	14.70	165–175	87	2.02
P-VP2	0.807	13.10	165–175	86	2
P-VP3	0.760	12.90	165–175	85	2

**Table 4:** Water absorption [%] vs. immersion time [h] (error < 1%).

Samples	72	168	240	336	408	504	576
L-PS1	3.19	4.88	7.63	10.43	13.16	15.79	15.82
L-PS2	3.30	7.57	11.13	13.10	16.75	20.07	20.16
L-PS3	3.32	8.87	13.31	15.54	19.91	24.02	24.02
H-PS1	3.22	4.92	7.84	10.81	13.72	16.19	16.29
H-PS2	4.19	6.42	10.03	13.73	17.31	20.77	20.82
H-PS3	4.26	11.44	17.23	19.96	25.72	31.01	31.03
H-VP1	3.48	6.08	10.09	12.30	16.15	18.18	18.18
H-VP2	3.84	8.88	13.70	14.78	18.13	21.98	22.07
H-VP3	5.37	8.20	13.07	18.01	22.86	26.99	27.15
P-VP1	2.25	6.05	9.11	10.56	13.61	16.41	16.42
P-VP2	2.93	5.69	8.67	12.28	15.22	18.56	18.56
P-VP3	4.39	6.71	10.69	14.74	18.70	22.08	22.21

**Table 5:** Transformer oil absorption [%] vs. immersion time [h] (error < 1%).

Samples	72	168	240	336	408	504	576
L-PS1	2.45	3.75	5.87	8.03	10.12	12.14	12.17
L-PS2	2.54	5.83	8.56	10.08	12.88	15.44	15.51
L-PS3	2.55	6.82	10.24	11.95	15.32	18.48	18.48
H-PS1	2.48	3.79	6.03	8.31	10.55	12.46	12.53
H-PS2	3.23	4.94	7.72	10.56	13.32	15.98	16.01
H-PS3	3.28	8.80	13.25	15.35	19.78	23.86	23.87
H-VP1	2.68	4.67	7.76	9.46	12.43	13.99	13.99
H-VP2	2.95	6.83	10.54	11.37	13.94	16.91	16.98
H-VP3	4.13	6.31	10.05	13.86	17.58	20.76	20.88
P-VP1	1.73	4.66	7.01	8.12	10.47	12.62	12.63
P-VP2	2.25	4.38	6.67	9.44	11.71	14.28	14.28
P-VP3	3.38	5.16	8.22	11.34	14.39	16.99	17.09

**Table 6:** Dielectric strength (error < 5%).

Samples	Dielectric Strength [kV/mm]
LDPEr	71
L-PS1	73
L-PS2	71
L-PS3	70
HDPEr	66
H-PS1	68
H-PS2	67
H-PS3	65
H-VP1	67
H-VP2	65
H-VP3	63
PPr	52
P-VP1	53
P-VP2	51
P-VP3	49

**Table 7:** Fire resistance performance.

Samples	Thermal Conductivity [W/(m × K)]	LOI [%]
LDPEr	0.332	18
L-PS1	0.346	19
L-PS2	0.353	20
L-PS3	0.367	21
HDPEr	0.446	19
H-PS1	0.451	20
H-PS2	0.464	20
H-PS3	0.476	21
H-VP1	0.453	20
H-VP2	0.467	21
H-VP3	0.485	22
PPr	0.245	19
P-VP1	0.258	20
P-VP2	0.271	21
P-VP3	0.287	22

## Data Availability

The original contributions presented in this study are included in the article. Further inquiries can be directed to the corresponding author.
